# γ-Aminobutyric Acid Alleviates Programmed Cell Death in Two *Brassica* Species Under Cadmium Stress

**DOI:** 10.3390/ijms26010129

**Published:** 2024-12-27

**Authors:** Zhong-Wei Zhang, Tao-Tao Dang, Xin-Yue Yang, Lin-Bei Xie, Yang-Er Chen, Ming Yuan, Guang-Deng Chen, Jian Zeng, Shu Yuan

**Affiliations:** 1 College of Resources, Sichuan Agricultural University, Chengdu 611130, China; zzwzhang@126.com (Z.-W.Z.); dttshzu@163.com (T.-T.D.); yang16970319@163.com (X.-Y.Y.); kkskado@163.com (L.-B.X.); doplin@gmail.com (J.Z.); 2 College of Life Science, Sichuan Agricultural University, Ya’an 625014, China; anty9826@163.com (Y.-E.C.); yuanming@sicau.edu.cn (M.Y.)

**Keywords:** cadmium stress, programmed cell death (PCD), γ-Aminobutyric acid (GABA), *Brassica* species, plant stress tolerance

## Abstract

Previous studies have demonstrated that γ-Aminobutyric acid (GABA) effectively alleviates heavy metal stresses by maintaining the redox balance and reducing the accumulation of reactive oxygen species (ROS). However, little is known about the role of GABA on programmed cell death (PCD) under Cd treatments in plants. The present study investigated the effects of GABA on Cd-induced PCD in two *Brassica* species, oilseed rape (*Brassica napus*, *Bn*), and black mustard (*Brassica juncea*, *Bj*). We observed that GABA significantly alleviated Cd-induced PCD by enhancing antioxidant systems, inhibiting chromatin condensation in the nucleus, and reducing DNA fragmentation under Cd stress. Moreover, GABA may not only reduce caspase-3-like activity by repressing gene expression, but also regulate transcription of PCD-related genes. *Bn* showed lower Cd accumulation and lower tolerance, with more pronounced PCD, compared with *Bj*. Our results provide new insights into the mechanism that GABA enhances Cd tolerance in plants.

## 1. Introduction

With the continuous development of industrial technology, heavy metal pollution has emerged as a major worldwide environmental concern that endangers human health and ecological balance [1]. Cadmium (Cd), as one of the most toxic heavy metals, destroys the permeability of plant cell membranes, inhibits mineral element absorption and transport, interferes with several physiological and metabolic functions, causes toxic effects on plants, impedes plant growth and development, and ultimately leads to cell death [2].

The use of exogenous regulators may effectively reduce the damage of metal stress on plants, improve plant growth and development, and increase crop yield and quality. γ-Aminobutyric acid (GABA) is a 4-carbon non-protein amino acid ubiquitous in plant cells [3] and has been widely studied for its function in regulating plant growth, metabolism, and biotic and abiotic stress tolerance [4,5]. Studies have found that GABA accumulates rapidly when plants are stressed [6,7], maintaining redox balance and preventing the accumulation of reactive oxygen species (ROS) under abiotic stress [8]; GABA also induced the accumulation of proline and polyamines (PAs) to improve plant resistance [9].

Cd promotes the accumulation of ROS in plant cells [10,11]. In the face of environmental stress, plant ROS can induce oxidative stress and even eventually lead to programmed cell death (PCD) [12]. ROS can also serve as a key signal molecule, involved in the perception of abiotic and biological stresses, the integration of environmental signals, and activation of the stress response networks, helping to establish a defense mechanism. Some ROS produced in the presence of different stressors play a role as a signal molecule with a cross tolerance mechanism and can adapt plants to other stresses [13,14]. The involvement of plant antioxidant systems, including the activity of antioxidant enzymes such as superoxide dismutase (SOD), catalase (CAT), and peroxidase (POD), as well as ascorbic acid (ASA) and glutathione (GSH), is essential for the reduction of oxidative damage caused by environmental stresses [13,14].

PCD is a gene regulatory process that orchestrates the clearance of specifically redundant cells, functioning as an organ defense mechanism against both abiotic and biotic stresses [15]. The cell morphological changes associated with PCD include cytoplasmic and nuclear condensation, DNA fragmentation, the formation of a characteristic DNA ladder, apoptotic body formation, and the swelling of subcellular organelles [16,17,18]. Additionally, non-morphological features of PCD include high-level accumulation of ROS, altered intracellular calcium levels, and the activities of caspase-like enzymes [19,20]. Among these, the caspase-3 protease from the caspase family plays a particularly prominent role in the process of cell apoptosis, and changes in its activity have become an important marker of cell apoptosis in animals [21]. Although caspase homologs are absent in plant genomes, caspase-like proteases have been identified in plants [22]. Meanwhile, researchers discovered that the proteasome subunit *PBA1* had a close relationship with caspase-3-like activity and played a key role in defensive mechanisms against bacterial infection [23]. Exogenous GABA has been reported to reduce oxidative damages and effectively delay PCD [24]. However, the relationship between GABA and Cd-stress-induced PCD in plants is still unclear.

As a hyper-accumulating plant of Cd, oilseed rape has great economic values [25]. Black mustard (*Brassica juncea* L.) has a high Cd accumulation ability and higher tolerance, but its biomass is lower, and the geographical distributions are limited. In contrast, the cabbage-type rape (*Brassica napus* L.) has characteristics of high yield and wide planting area [26]. Researchers have shown that exogenous GABA could alleviate the inhibition of Cd stress on plant growth effectively, reduce the accumulation of Cd, and thereby reduce the toxic effects of Cd stress on plants [27,28]. However, it remains to be investigated whether there are differences in the effects of GABA on PCD in two oilseed rape species with different Cd tolerances. We hypothesized that exogenous GABA can alleviate Cd-induced PCD by reducing Cd accumulation, inhibiting production of ROS, activating antioxidant defense system, and regulating gene expression and protease activities associated with PCD. Based on this hypothesis, the objective of this study was to explore the effects of exogenous GABA on PCD of different Cd-tolerant oilseed rape species under Cd stress and to reveal the underlying molecular mechanisms.

## 2. Results

### 2.1. Effects of Exogenous GABA on Cd Content and GABA in Two Brassica Species

**Figure 1:**
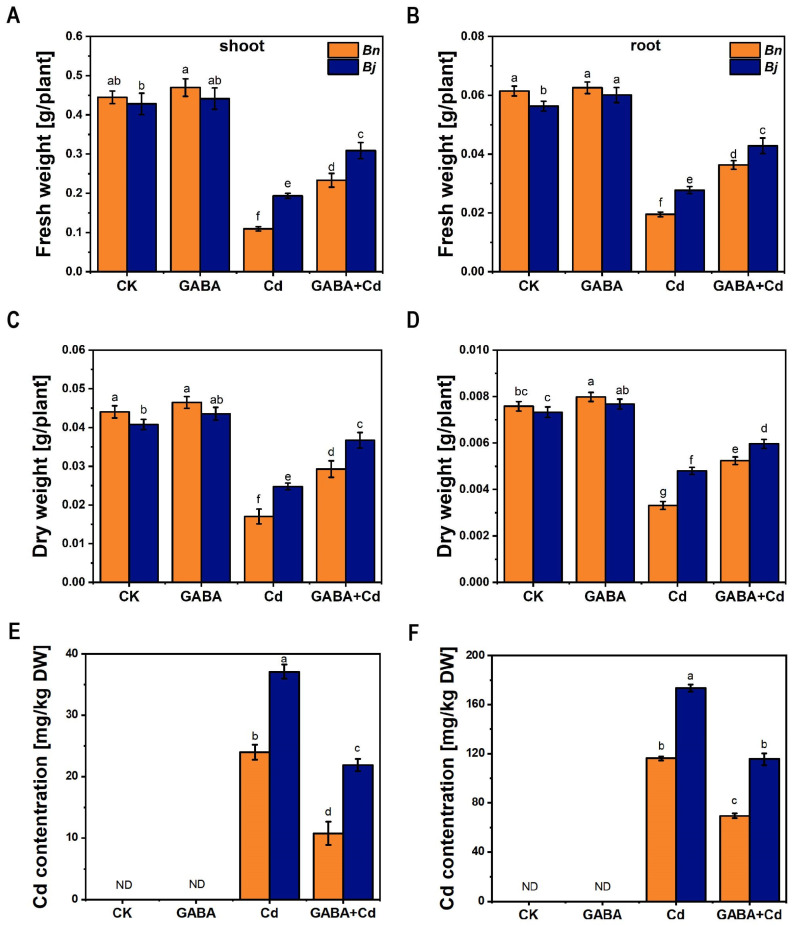
Effects of exogenous GABA on biomass and Cd content of two *Brassica* species seedlings under Cd stress for 7 days. Fresh weight in shoots (**A**) and roots (**B**). Dry weight in shoots (**C**) and roots (**D**). Cd content in shoots (**E**) and roots (**F**). *Bn*, *Brassica napus*; *Bj*, *Brassica juncea*; CK, control; GABA, 5 mM GABA; Cd, 50 µM CdCl_2_; GABA + Cd, 5 mM GABA and 50 µM CdCl_2_ co-treatment. DW: dry weight; ND: Not detected. Error bars show standard deviations (*n* = 3). Different lowercase letters indicate significant differences at a level of *p* ≤ 0.05.

As shown in Figure 1A–D, the biomass of both *Brassica* species seedlings was significantly reduced under Cd treatment; however, compared with *Bn*, the biomass of *Bj* was more significantly declined under the Cd stress. The biomass of shoots and roots of both *Brassica* species increased significantly after exogenous GABA addition.

Consistent with previous reports [26,29], the accumulation levels of Cd in *Bj* were always higher than those in *Bn*. Compared with Cd-alone treatment, the addition of GABA decreased Cd levels by 40–55% and 25–33% in *Bn* and *Bj*, respectively (Figure 1).

Under Cd stress, GABA contents of two *Brassica* species increased significantly (Supplementary Figure S1A,B), and the GAD activity also increased significantly (Supplementary Figure S1C,D). Elevated GAD activity promotes GABA synthesis, which is a key adaptation mechanism in plants responding to environmental stresses [30]. The degradation of GABA is mainly through the GABA-hunt process, in which GABA transaminase (GABA-T) catalyzes the conversion of GABA to succinic semialdehyde (SSA). Under the Cd stress, the GABA-T activity of two *Brassica* species decreased significantly (Supplementary Figure S1E,F), which reduced the degradation of GABA and enhanced the accumulation of endogenous GABA in cells. Compared with *Bj*, *Bn* had a little higher content of GABA but a little lower level of GABA-T activities (Supplementary Figure S1).

### 2.2. Exogenous GABA-Enhanced Cd Tolerance

**Figure 2:**
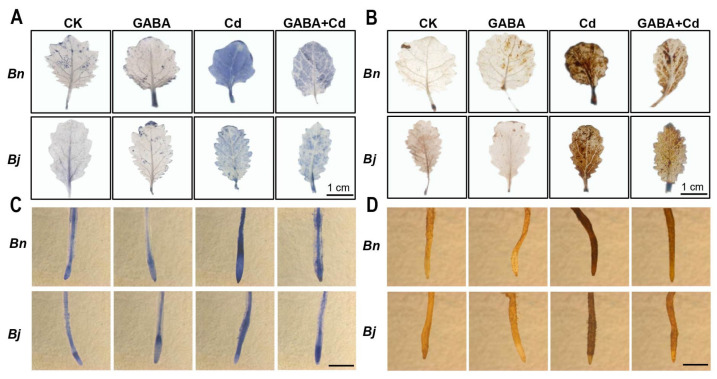
H_2_O_2_ and O_2_^−^ staining in *Brassica* species seedlings. O_2_^−^ (**A**) and H_2_O_2_ (**B**) staining in shoots, Bars = 1 cm. O_2_^−^ (**C**) and H_2_O_2_ (**D**) staining in roots, Bars = 500 µm. *Bn*, *Brassica napus*; *Bj*, *Brassica juncea*; CK, control; GABA, 5 mM GABA; Cd, 50 µM CdCl_2_; GABA + Cd, 5 mM GABA and 50 µM CdCl_2_ co-treatment.

ROS staining showed differences in H_2_O_2_ and O_2_^−^ contents in leaves and roots (Figure 2 and Supplementary Figure S2). After Cd stress, the *Bn* seedlings accumulated more H_2_O_2_ and O_2_^−^ than *Bj* seedlings. However, GABA + Cd co-treatment greatly decreased ROS accumulation. Nevertheless, the alleviating effect of GABA on *Bj* was relatively small compared with that of *Bn*. The MDA contents showed a similar pattern with ROS staining (Supplementary Figure S2). GABA treatments increased antioxidant enzyme activities and non-enzymatic antioxidant levels (reduced glutathione and ascorbic acid), which were more induced in *Bj* than in *Bn* (Supplementary Figures S3–S5).

### 2.3. Effect of GABA on Cell Death in Two Brassica Species Under Cd Stress

**Figure 3:**
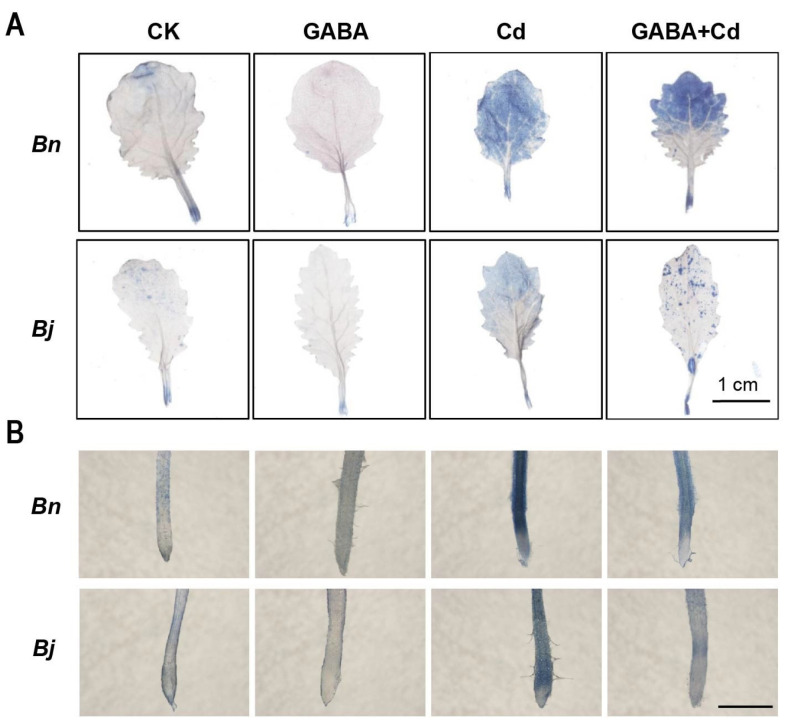
Effect of GABA on cell death under Cd stress for 7 days. (**A**) Evans blue staining in shoots, Bars = 1 cm; (**B**) Evans blue staining in roots, Bars = 500 µm. *Bn*, *Brassica napus*; *Bj*, *Brassica juncea*; CK, control; GABA, 5 mM GABA; Cd, 50 µM CdCl_2_; GABA + Cd, 5 mM GABA and 50 µM CdCl_2_ co-treatment.

Cd stress promotes the accumulation of intracellular ROS and therefore induces cell death [31]. Evans blue is a cell-reactive dye that stains the cell walls of dead cells [32]. As shown in Figure 3, Cd treatment significantly induced cell death, with strong blue staining observed in the plant leaves and roots. Notably, the degree of staining in *Bn* was more pronounced than in *Bj*. Furthermore, application of GABA under the Cd treatment reduced the degree of Evans blue staining, suggesting that GABA could effectively reduce cell death induced by Cd stress.

### 2.4. Cd-Induced Cell Death in Two Brassica Species Was Driven by a PCD Process

**Figure 4:**
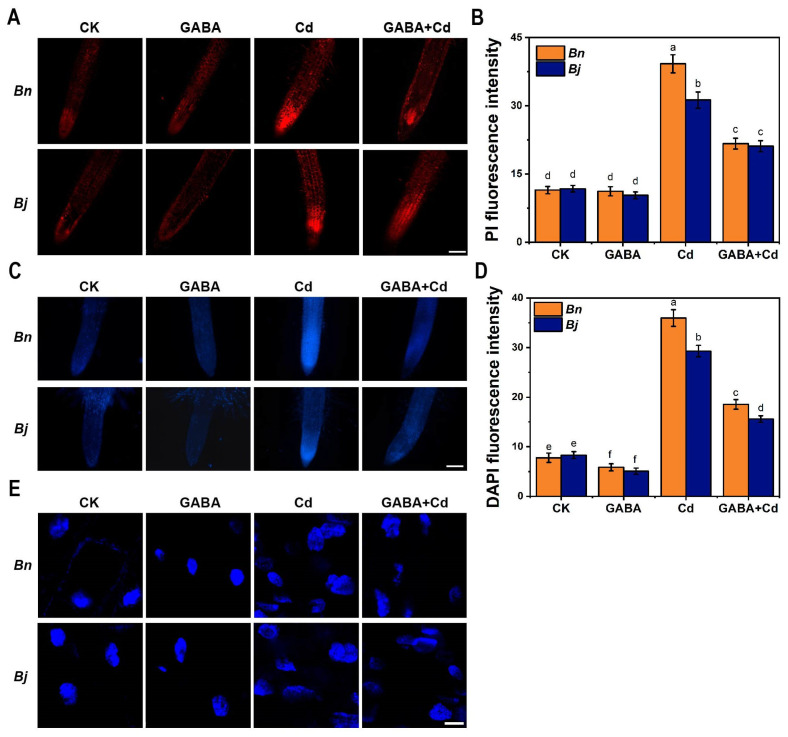
Effects of GABA on PCD in two *Brassica* species under Cd stress for 7 days. (**A**) PI staining in roots, Bars = 200 µm. (**B**) Quantitative analysis of the PI fluorescence. (**C**) DAPI staining in roots, Bars = 200 µm. (**D**) Quantitative analysis of the DAPI fluorescence. (**E**) DAPI staining of cell nucleus in root tips, Bars = 20 µm. *Bn*, *Brassica napus*; *Bj*, *Brassica juncea*; CK, control; GABA, 5 mM GABA; Cd, 50 µM CdCl_2_; GABA + Cd, 5 mM GABA and 50 µM CdCl_2_ co-treatment. Error bars show standard deviations (*n* = 3). Different lowercase letters indicate significant differences at a level of *p* ≤ 0.05.

We chose several diagnostic markers of PCD to investigate whether Cd-caused cell death is a kind of PCD. Propidium iodide (PI) is a DNA-binding dye that is membrane-impermeable and is often used to quantify cell death [33]. As shown in Figure 4A, the fluorescence signals of PI in the root tip of both *Brassica* species were significantly enhanced under Cd stress, especially in the root tips of *Bn* (Figure 4B). However, the fluorescence signals at the root tips decreased significantly after the GABA + Cd co-treatment (Figure 4), indicating that GABA could protect the integrity of the apical cell membrane and thus alleviate Cd stress.

4′,6-diamidino-2-phenylindole (DAPI), a fluorescent dye that binds strongly to DNA, is commonly used to observe nuclear morphological changes in apoptosis assays [33]. According to DAPI staining (Figure 4C,D), in the absence of Cd stress, the nuclei of two *Brassica* species showed regular oval and uniform blue fluorescence, indicating the integrity of the nucleus. After Cd treatment, the DAPI fluorescence signal was significantly enhanced, the chromatin condensed, and the nucleus appeared stretched, with an irregular granular staining. In contrast, more round and uniformly stained nuclei can be found, and the DAPI fluorescence signal was significantly reduced under the GABA + Cd co-treatment, implying that GABA relieved Cd-induced PCD and reduced nuclear damages.

### 2.5. GABA Relieved Cd-Stress-Induced DNA Fragmentation

**Figure 5:**
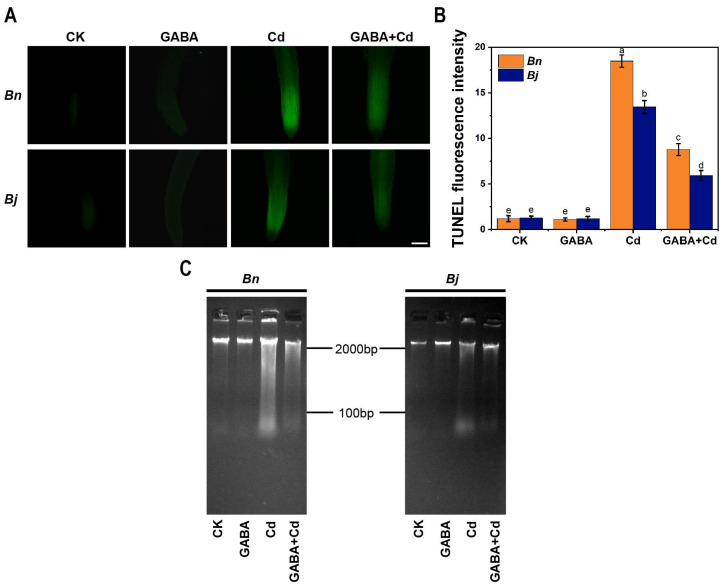
DNA fragmentation visualized by DNA laddering and TUNEL assay. (**A**) TUNEL staining in roots, Bars = 200 µm. (**B**) Quantitative analysis of the TUNEL fluorescence. (**C**) DNA laddering of genomic DNA separated by 2% agarose gel electrophoresis. *Bn*, *Brassica napus*; *Bj*, *Brassica juncea*; CK, control; GABA, 5 mM GABA; Cd, 50 µM CdCl_2_; GABA + Cd, 5 mM GABA and 50 µM CdCl_2_ co-treatment. Error bars show standard deviations (*n* = 3). Different lowercase letters indicate significant differences at a level of *p* ≤ 0.05.

A typical molecular hallmark of PCD is DNA fragmentation, the extent of which can be examined by the Terminal deoxynucleotidyl transferase-mediated deoxy-uracil nick end labeling (TUNEL) staining (free 3′-OH ends of DNA can be fluorescently labeled) [34]. As can be seen in Figure 5A,B, the TUNEL-positive signals were significantly enhanced after the Cd treatment, and the fluorescence intensity of the *Bn* root tip was significantly higher than that of *Bj*. However, the addition of GABA under the Cd stress significantly attenuated TUNEL-positive signals at the root tips of both *Brassica* species.

Meanwhile, PCD induces the production of endonuclease, leading to the degradation and breakage of nuclear DNA between nucleosomes, producing oligonucleotide body fragments, which can be observed in gel electrophoresis [35,36]. As shown in Figure 5C, the DNA bands were clear and intact under CK and GABA treatment without Cd stress. Contrastingly, an evident DNA laddering could be observed after the Cd treatment, and that of *Bn* was more obvious than that of *Bj*. Compared to the Cd treatment alone, the DNA fragmentation was relieved in the GABA + Cd co-treatment (Figure 5C). In conclusion, GABA could attenuate PCD by inhibiting DNA degradation.

### 2.6. Effects of GABA on Caspase-3-like Activity and PBA1 Expression Under Cd Stress

**Figure 6:**
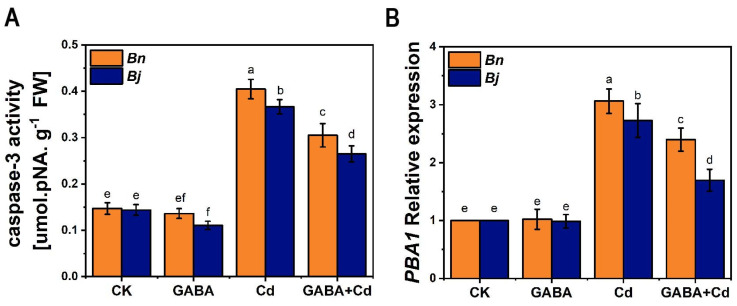
Effect of GABA on the caspase-3-like activity and the expression of *PBA1* in *Brassica* seedlings under Cd stress for 7 days. (**A**) Caspase-3-like protease activity. (**B**) *PBA1* expression. *Bn*, *Brassica napus*; *Bj*, *Brassica juncea*; CK, control; GABA, 5 mM GABA; Cd, 50 µM CdCl_2_; GABA + Cd, 5 mM GABA and 50 µM CdCl_2_ co-treatment. The gene expression levels of the control seedlings (CK) were normalized to 100%. Error bars show standard deviations (*n* = 3). Different lowercase letters indicate significant differences at a level of *p*≤ 0.05.

Caspase-3, a key enzyme in PCD, serves as its executioner, with its activation signifying an irreversible PCD process. While plants have no caspase-3 homologs, 20S proteasome subunit PBA1 works as a caspase-3-like protease in plant PCD instead [37]. Here, we explored the mechanism of Cd-induced PCD by measuring caspase-3-like activities and PBA1 expression. Figure 6 shows that Cd treatment induced a 2–3 times increase in caspase-3-like activity and PBA1 expression. Under the Cd stress and GABA + Cd co-treatment, PBA1 expression was significantly higher in *Bn* than in *Bj*. Exogenous GABA could significantly reduce both caspase-3-like activity and PBA1 expression under stress. Therefore, we presumed that GABA may regulate PCD by modulating caspase-3-like activity at the transcriptional level.

### 2.7. The Role of GABA in the Regulation of Gene Expression Associated with PCD

**Figure 7:**
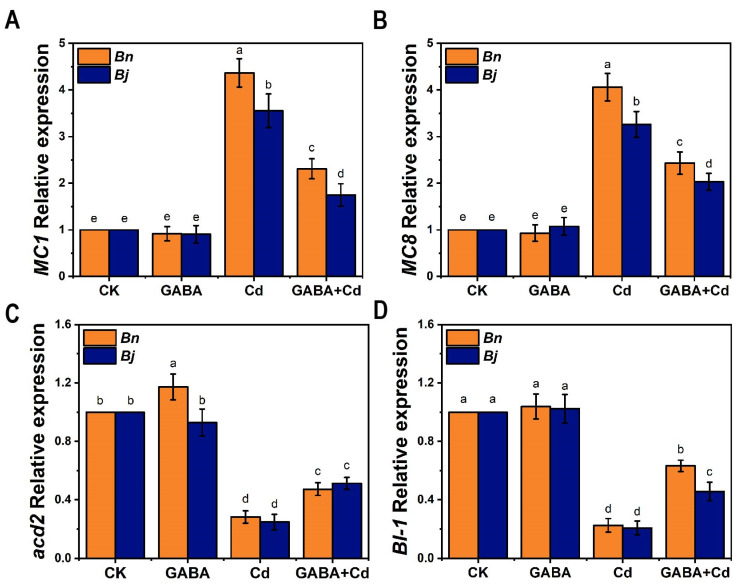
Effects of GABA on the expression of genes involved in PCD in two *Brassica* species under Cd stress for 7 days. Four representative genes, *MC1* (**A**), *MC8* (**B**), *Acd2* (**C**), and *BI-1* (**D**) were studied. Detection was performed using quantitative real-time analysis under different treatment conditions. The expression levels of the control seedlings (CK) were normalized to 100%. *Bn*, *Brassica napus*; *Bj*, *Brassica juncea*; CK, control; GABA, 5 mM GABA; Cd, 50 µM CdCl_2_; GABA + Cd, 5 mM GABA and 50 µM CdCl_2_ co-treatment. Error bars show standard deviations (*n* = 3). Different lowercase letters indicate significant differences at a level of *p* ≤ 0.05.

To further explore the mechanism of Cd-induced PCD, we analyzed the gene expression pattern associated with PCD. We hypothesized that GABA may influence the transcription of key PCD-related genes, thereby affecting the progression of PCD under Cd stress. A large number of genes have been identified in plant PCD, including programmed cell death (*PDCD*) genes, metacaspase (*MC*) genes, vacuolar processing enzyme (*VPE*) genes [31], accelerated cell death (*ACD*) genes [38], uncoupling protein (*UCP*) genes [39], and the Bax inhibitor-1 (*BI-1*) gene [36,38,40]. The results of RT-qPCR are shown in Figure 7, where Cd treatment induced expression of two PCD enhancer genes, *MC1* and *MC8*, while suppressing expression of two PCD inhibitor genes, *Acd2* and *BI-1*, compared with the control seedlings. It was worth noting that the expression of *MC1* and *MC8* was significantly suppressed after the GABA + Cd co-treatment, while *Acd2* and *BI-1* expression were significantly increased. Under the Cd stress and GABA + Cd co-treatment, MC1 and MC8 expression was significantly higher in *Bn* than in *Bj*. The results suggested that GABA could alleviate the toxicity of Cd by regulating transcription of genes associated with PCD.

## 3. Discussion

Cadmium (Cd) is a non-essential and toxic heavy metal and has become a common pollutant in agricultural soils. Cd^2+^ ions are highly soluble and migratory and are easily absorbed by plants [29,41]. Upon absorption by the plant root cortex, Cd reaches the xylem through the exoplastid or symplast pathway. The majority of the Cd complexes remain in the root, with only a minor fraction being transported into the plant shoots. GABA plays a multitude of physiological roles within plants [42]. Under various stress conditions, GABA has been shown to produce a transient release of Ca^2+^, which binds to cell surface receptors. The activation of Ca^2+^ stimulates GAD activity, thereby increasing the levels of GABA within the cell [43]. According to Lee et al. [44], GABA synthesized by GAD can enhance the tolerance of plant cells to various stresses. Jalil et al. [45] also demonstrated that GABA-T is a key factor in reducing oxidative damage and improving stress resistance in plants. In our study, GABA content increased under Cd treatment (Supplementary Figure S1), and the exogenous addition of GABA reduced Cd accumulation in *Brassica* seedlings (Figure 1). This is consistent with previous studies, which further prove that the application of exogenous GABA can reduce the absorption and accumulation of Cd in plants, thus improving the tolerance of Cd [9,27,28].

Under stress conditions, excessive accumulation of ROS can initiate a series of oxidative stress responses. These responses include cell membrane peroxidation, alterations in protein structure, a decline in enzyme activity, and DNA damage [46,47,48,49]. Such oxidative stress can ultimately result in cell death. This phenomenon has been widely observed across various plant species, including cucumber [50], tomato [51], *Arabidopsis* [52], and tobacco [53]. Our study further revealed that Cd-caused cell death in *Brassica* seedlings is a kind of PCD accompanied by typical PCD features, such as chromatin condensation in the nucleus and DNA fragmentation (Figure 4 and Figure 5).

GABA has been suggested to play a key role in antioxidant defense mechanisms in plant cells [54]. Studies have shown that the addition of exogenous GABA could effectively decrease the accumulation of ROS by activating a range of antioxidant enzymes [55,56]. Activation of these enzymes helps to remove excess ROS and relieve oxidative stress, thereby protecting plant cells from oxidative damage and reducing the occurrence of PCD [33].

At the molecular level, Cd stress is able to interfere with the expression of proteins and genes associated with PCD, thus leading to the disruption of cell defense mechanisms [57,58]. Members of the caspase family play central roles in animal cell apoptosis, during which they are often co-expressed and activated [59]. Although the genome of land plants does not contain typical caspases, caspase-like proteases have also been found to play a key role in plant PCD [60]. In particular, the caspase-3-like enzyme is considered a key executor in the process of apoptosis [60]. In Arabidopsis suspension cells, 100 μM Cd treatment was found to increase caspase-3-like activities [61]. Hatsugai et al. [23] indicated that the proteasome subunit *PBA1* works as the caspase-3-like enzyme in *Arabidopsis.* Cai et al. [62] further indicated that *PBA1* may have a caspase-3-like activity and play a role in *Arabidopsis* PCD induced by the endoplasmic reticulum (ER) stress. Recently, Huai et al. [37] proposed that *PBA1* is a widely conserved component acting as a key enzyme in plant PCD, potentially involved in nuclear degradation upon PCD. In this report, we found a positive correlation between the expression of *PBA1* and caspase-3-like activity. Meanwhile, GABA can alleviate PCD by inhibiting Cd-stress-induced *PBA1* expression and reducing caspase-3-like activity (Figure 6), further supporting the potential role of *PBA1* in Cd-induced PCD regulation.

Transcriptome reprogramming also plays a crucial role in the development of PCD. Currently, multiple genes associated with PCD have been identified in plants. For example, the Metacaspase (*MC*) family is a family of genes distantly related to caspase found in plants, fungi, and protozoa. In the *Arabidopsis thaliana* genome, the *AtMC1* and *AtMC8* genes have been shown to be positive regulators in PCD due to biotic or abiotic stresses [36,63]. Pattanayak et al. [38] found that the *accelerated cell death 2* (*Acd2*) gene could inhibit the burst of ROS in mitochondria and negatively regulate cell death in Arabidopsis cells. In addition, Bax inhibitor-1 (*BI-1*), an evolutionarily conserved protein localized in the ER, is capable of inhibiting the development of PCD [40]. Over-expression of *BI-1* has been observed to suppress the cell death induced by Bax, pathogens, or other environmental factors [64]. The application of exogenous GABA could alleviate Cd-induced PCD by reducing the expression of PCD positive regulators *MC1* and *MC8* and increasing the expression levels of negative regulators *Acd2* and *BI-1* (Figure 7).

**Figure 8:**
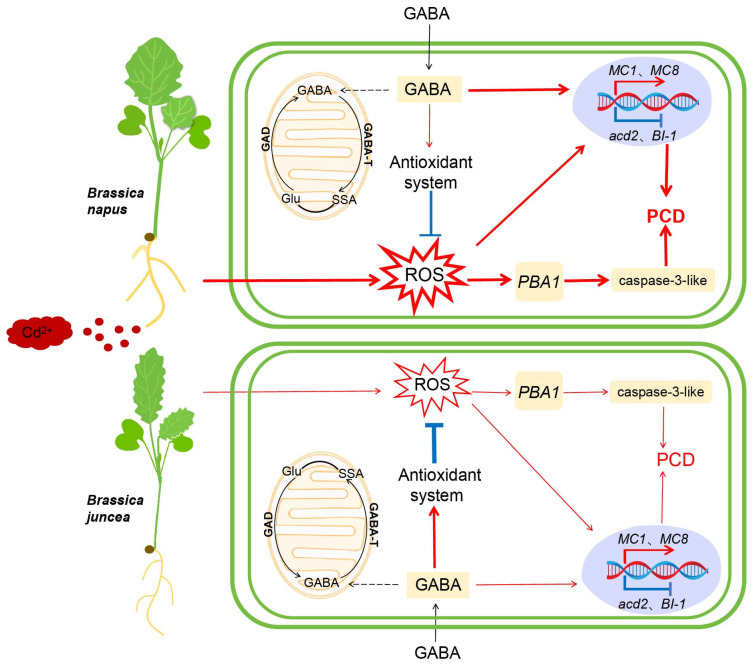
Schematic diagram of the mechanism that GABA relieves Cd-stress-induced PCD in two *Brassica* species. Cd stress induces the production of ROS, which then cause oxidative stress, eventually leading to PCD. Exogenous addition of GABA can increase the GABA content in plants and thus exert a protective effect. GABA could alleviate PCD by enhancing antioxidant enzyme activity, thus reducing ROS accumulation and further inhibiting *PBA1* expression to reduce caspase-3-like protein activity. On the other hand, GABA is involved in regulating the expression of PCD-related genes. GAD, glutamate decarboxylase; GABA-T, GABA transaminase; SSA, succinic semialdehyde; acid; Glu, glutamate. The thickness of the line represents the magnitude of the change.

Among *Brassica* species, *Brassica juncea* (*Bj*) exhibits superior Cd accumulation and tolerance compared to *Brassica napus* (*Bn*) [25,26]. However, the underlying molecular mechanism responsible for this difference is still largely unknown. *Bn* accumulated less Cd compared to *Bj* but showed higher levels of ROS and exhibited a more pronounced PCD upon Cd exposure. Although *Bn* had a slightly higher GABA content than *Bj*, the transcript levels of *PBA1*, *MC1*, and *MC8* were more elevated in *Bn* compared to *Bj* under the Cd stress. Transcriptional activation might be one of the reasons for the different PCD performance between two *Brassica* species (Figure 8).

Plants’ resistance to heavy metal stress and other biotic stresses can be interconnected [13,14]. Previous studies indicated that *Bj* was more tolerant to drought, ozone, and heat stress than *Bn* [65,66,67]. More activated antioxidant systems in *Bj*, induced by ROS signals or GABA signals, may be one of the reasons. Some plants are very resistant to cadmium, and this is connected to their photosynthetic metabolism and also their transportation of heavy metals within the plant [68,69]. Some plants can even accelerate their gas exchange when exposed to Cd [70]. Reaction centers of photosystem II of *Bj* were more photoprotected and hence more active than those of *Bn* under the salt stress [71,72]. On the other hand, *Bj* may have higher activities of some heavy metal transporters, such as natural-resistance-associated macrophage proteins (NRAMPs), ATP-binding cassette (ABC), heavy metal ATPase (HMA), cation diffusion facilitator (CDF), yeast cadmium factor 1 (ycf1), and so on [73,74]. Thus, higher Cd accumulation and higher tolerance in *Bj* may be attributed to multiple reasons besides attenuated PCD.

## 4. Materials and Methods

### 4.1. Plant Materials and Treatments

The seeds of cultivar Zhongshuang 11 of *Brassica napus* (*Bn*) and cultivar Gaoxin 13 of *Brassica juncea* (*Bj*) were surface sterilized with 1% sodium hypochlorite for 10 min and vernalized at 4 °C for 24 h. Then, the seeds of two *Brassica* species were placed evenly on wet filter paper and cultured at 21 °C with constant temperature and humidity for 3–4 days. The plants were then transferred to a modified Hoagland nutrient solution in a green-house chamber with a 14 h day/10 h night at 24 °C/20 °C cycle, relative humidity 60–75%, and light intensity of 120 µmol m^−2^ s^−1^. At one week after germination, the seedlings were treated with 50 µmol/L CdCl_2_ [26] and 5 mmol/L GABA [28], and the samples were collected after 7 days of treatments.

For GABA and GABA + Cd treatments, 5 mmol/L GABA solution was sprayed to the whole seedlings every 2 days [45]. The control group was sprayed with distilled water every 2 days. Each treatment was performed in triplicate.

### 4.2. Determination of Cd Content

A total of 0.2 g of roots or shoots samples were digested in a mixture of acid containing HClO_4_/HNO_3_ (*v/v* = 1/4) and then diluted into 50 mL. Inductively coupled plasma mass spectrometry (Agilent 7900 ICP-MS, Santa Clara, USA) was adopted to quantify the total Cd content [75].

### 4.3. Determination of GABA Content and Related Enzymatic Activities

GABA content was determined with reference to the method of Wang et al. [76]. The O.D. value was measured at 645 nm by a Schimadzu UV-1800 spectrophotometer. Glutamate decarboxylase (GAD) activity was measured based on the method of Yogeswara et al. [77]. The absorbance was determined at 630 nm by UV spectrophotometry. GABA transaminase (GABA-T) activity was measured with reference to the method of Jalil et al. [45]. The O.D. value at 340 nm was recorded using a Schimadzu UV-1800 spectrophotometer.

### 4.4. ROS Staining and Quantification of Oxidative Damages

The levels of superoxide (O_2_^−^) and hydrogen peroxide (H_2_O_2_) in shoots and roots were measured using nitro blue tetrazolium (NBT) and 3,3-diaminobenzidine (DAB), respectively [29,78]. After Cd/GABA treatments, leaves and roots were dyed with 0.8 mg/mL NBT or 2.4 mg/mL DAB for two hours. Samples were then bleached in 80% ethanol. The contents of malondialdehyde (MDA) were measured by the hiobarbituric acid-reactive substances (TBARS) method [79].

### 4.5. Determination of Antioxidant Enzyme Activities

Fresh samples were homogenized using a pre-chilled mortar and pestle with 5 mL of a proprietary extractant solution, which consisted of 50 mM Tris-HCl buffer and 20% glycerin, 1 mM ascorbic acid (AsA), 1 mM dithiothreitol (DTT), and 1 mM glutathione (GSH). Following homogenization, the mixture was centrifuged at 20,000× *g* for 20 min at 4 °C. The supernatant, which had separated, was then carefully collected for subsequent testing. The measurement of superoxide dismutase (SOD) was determined according to Liu et al. [80]. Peroxidase (POD) activities were conducted according to Verma and Mishra [81]. The measurements of catalase (CAT) activities were conducted according to Esfandiari et al. [82].

A total of 0.2 g of root or shoot samples were re-suspended in 0.5 mL 5% 5-sulfosalicylic acid and 2 mL phosphate buffer (PBS; pH 7.4) and sonicated for 15 min. Quantification of reduced glutathione and oxidized glutathione (GSH and GSSG) was performed according to Cao et al. [83]; quantification of ascorbic acid (AsA) and dehydroascorbic acid (DHA) was performed according to Kampfenkel et al. [84].

### 4.6. Detection of Cell Death

Evans blue staining has been widely used as an indicator of cell death. The seedling leaves and roots (2 cm) were stained for 48 h with 0.25% (*w/v*) Evans Blue for 15 min, washed with water for 15 min, and then observed and photographed under a microscope [34].

### 4.7. Propidium Iodide (PI) Staining

To measure cell death, the root tip was stained with 10 μg/mL PI for 20 min and washed in water twice before being viewed with a confocal laser scanning microscope (LeicaSP2; Buffalo Grove, IL, USA). The excitation wavelength was set at 560 nm, with an emission at 580–680 nm [33].

### 4.8. Terminal Deoxynucleotidyl Transferase-Mediated Deoxy-Uracil Nick End Labeling (TUNEL) Assay

The One Step TUNEL Apoptosis Assay Kit (Beyotime, Shanghai, China) was used to investigate nuclear DNA degradation. The root tip was incubated with TUNEL detection solution for 40 min at 37 °C in the dark. The root tip was then rinsed three times with 10 mM phosphate buffer saline (pH 7.4) and examined using the confocal laser scanning microscope. The excitation wavelength was set at 488 nm, and the emission at 530 nm [33].

### 4.9. 4′,6-Diamidino-2-phenylindole (DAPI) Staining

The root tip was stained with 1.2 mg/LDAPI (dissolved at 10 mmol/L Tris-HCl, pH 7.4) for 15 min, then washed twice with water and examined by the confocal laser scanning microscope under excitation at 345–375 nm [33].

### 4.10. Caspase-3-like Protease Activity Determination

The caspase-3-like test kit (Solarbio, Beijing, China) was utilized in compliance with the guideline provided by the manufacturer. In a 96-well microtiter plate, 40 μL of extract was incubated with 60 µL reaction buffer (5 µL caspase-3 substrate, 2 mM) to determine caspase-3 activities. The lysates were incubated for 4 h at 37 °C. The mixtures were determined at 405 nm with an ELISA micro-plate reader [39].

### 4.11. DNA Extraction and Analysis

The DNA was extracted in 500 µL 0.2% cetyltrimethylammonium bromide (CTAB) buffer. After incubating at 55 °C for 30 min, 500 µL of chloroform: isoamyl alcohol was added at a ratio of 24:1 (*v/v*). Finally, the DNA was precipitated using cold isopropanol. After washing with 70% ethanol, the precipitates were dissolved in 50 µL of deionized water at 55 °C. The isolated genomic DNA was put onto a 2% agarose gel, and the gel images were captured after electrophoresis [39].

### 4.12. Quantitative Real-Time PCR Analysis

The expression levels of four PCD-related genes were determined by quantitative real-time PCR analysis. The cDNA was amplified using SYBR Premix Ex Taq (Takara Biomedical Technology Co., Ltd., Dalian, China). The threshold (Ct) value, defined as the PCR cycle that initially detected a statistically significant increase in reporter fluorescence, was used to estimate the initial copy number of the target gene [85]. Three biological replicates were performed for each sample. The *ACTIN* gene was used as an internal control. The expression level of *Brassica* seedlings without Cd/GABA treatment was normalized to 100%. All primers are included in Supplementary Table S1.

### 4.13. Statistical Analysis

All experiments were performed at least three times, and mean values are presented with standard deviations (*n* ≥ 3). Student’s *t* tests were used for comparison between different treatments. The differences were judged statistically significant at *p* < 0.05.

## 5. Conclusions

Cd accumulation in oilseed rape leads to an oxidative stress, characterized by the production of ROS, which in turn triggers PCD. GABA alleviates PCD of *Brassica* species under Cd stress by reducing the accumulation of Cd, maintaining cellular osmotic balance, enhancing antioxidant enzyme activity, and reducing PCD features. On the other hand, exogenous GABA may reduce the activity of caspase-3-like proteins by inhibiting the expression of *PBA1* and participating in regulating the expression of PCD-related genes. Among *Brassica* species, *Brassica napus* exhibits more pronounced PCD response, potentially due to its lower antioxidant enzyme activities and non-enzymatic antioxidant levels, as well as higher PCD-enhancer gene transcript levels compared to *Brassica juncea*.

## Data Availability

All data generated or analyzed during this study are included in this published article and its Supplementary Information file.

## Supplementary Materials

The following supporting information can be downloaded at:https://www.mdpi.com/article/10.3390/ijms26010129/s1.
