# Supplementary material for: γ-Aminobutyric Acid Alleviates Programmed Cell Death in Two Brassica Species Under Cadmium Stress
Source: Int J Mol Sci. 2024 Dec 27;26(1):129. doi: 10.3390/ijms26010129 (PMC11720724; doi:10.3390/ijms26010129)
Supplement: Supplementary file 1 [file ijms-26-00129-s001.zip › ijms-3361111-supplementary.pdf]

Supplementary Table S1. Primers used for qRT-PCR analysis in the present study.

Gene name	Forward primer	Reverse primer
BnPBA1	5 ′-GCCTGCCACTGTAAAGGTCTC-3′	5 ′-GGATGATTGTTTGTTTGCATCACAT-3′
BnMC1	5 ′-CCCTCAAGCATCTACGCTCC-3′	5 ′-AGACGTGATCTTCGACAGCG-3′
BnMC8	5 ′-ACCGGATGCAGAAATGCCTA-3′	5 ′-ATTCGGGCTTGGGTCTTCAC-3′
BnAcd2	5 ′-TCCCTGTAACCTCCCTCCAG-3′	5 ′-TCGAGAACCTCCTTAGCAGC-3′
BnBI-1	5 ′-GGAGCTTACCTCCATGTGCT-3′	5 ′-TCACCGAGGTGTGCCTTTTC-3′
ACTIN	5 ′-TGGGTTTGCTGGTGACGATG-3′	5′-GATCGAGACGGAGGATAGCG-3′

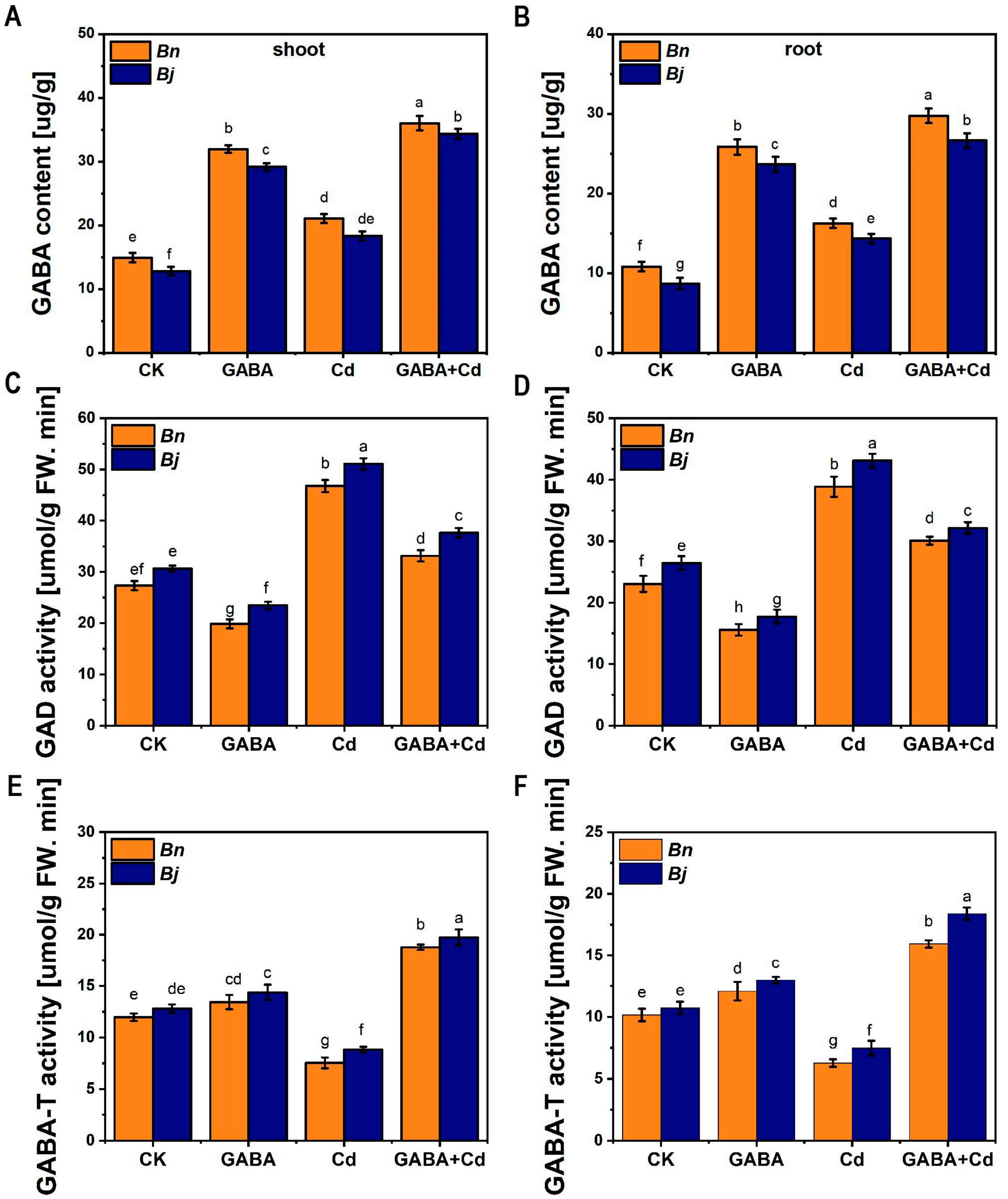

Supplementary Figure S1. Effects of exogenous GABA on GABA content and related enzyme activities in GABA metabolism in *Brassica* seedlings under Cd stress. GABA content in shoots (**A**) and roots (**B**). GAD activity in shoots (**C**) and roots (**D**). GABA-T activity in shoots (**E**) and roots (**F**). *Bn*, *Brassica napus*; *Bj*, *Brassica juncea*; CK, control; GABA, 5 mM GABA; Cd, 50 μM $CdCl_2$; GABA + Cd, 5 mM GABA and 50 μM $CdCl_2$ co-treatment. FW: fresh weight. Error bars show standard deviations (*n* = 3). Different lowercase letters indicate significant differences at a level of $P \leq 0.05$.

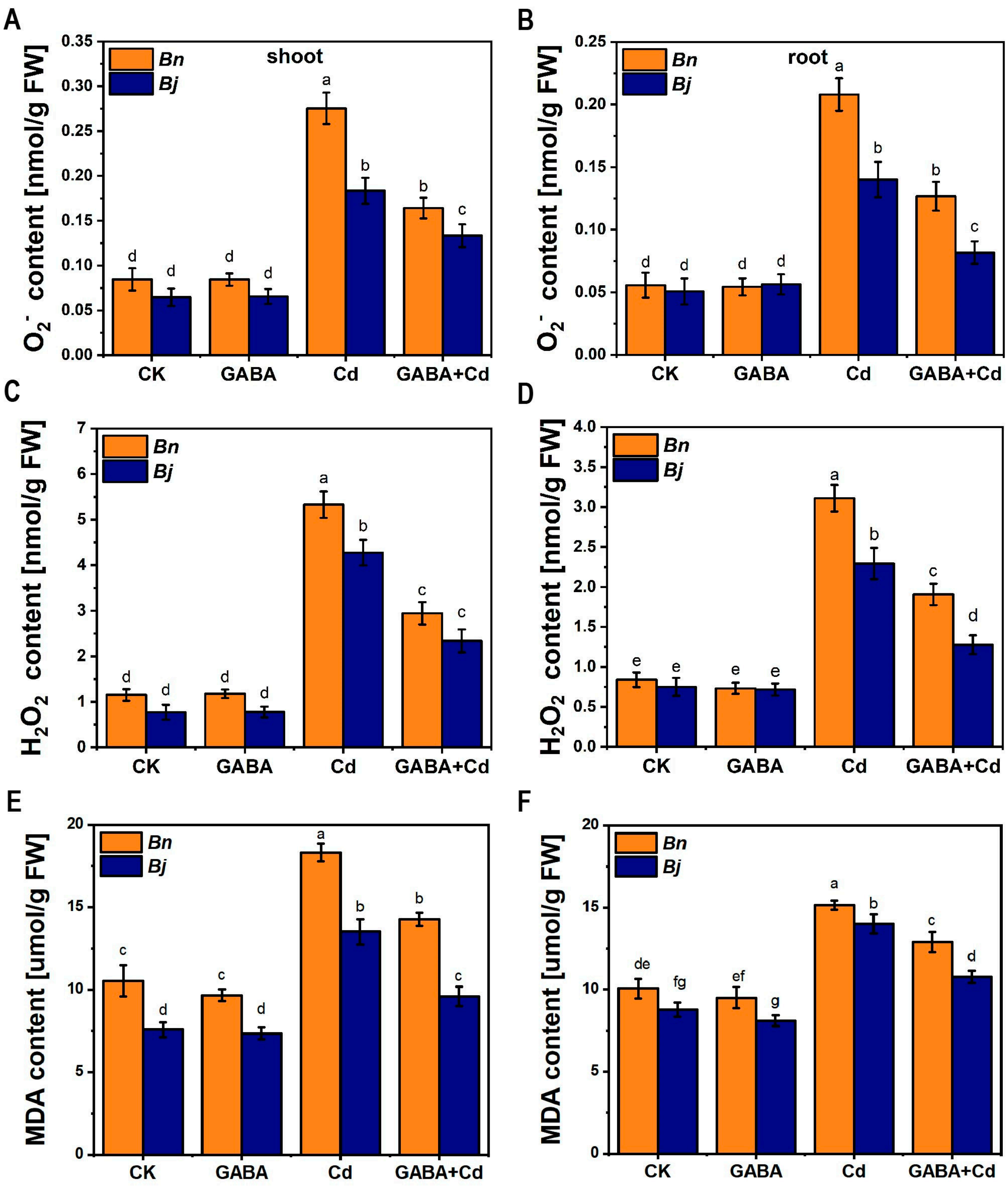

Supplementary Figure S2. Effects of GABA on the content of H_2O_2, $O_2{\cdot}^-$ and malondialdehyde (MDA) in *Brassica* seedlings under Cd stress. $O_2{\cdot}^-$ content in shoots (**A**) and roots (**B**). H_2O_2 content in shoots (**C**) and roots (**D**). *Bn*, *Brassica napus*; *Bj*, *Brassica juncea*; CK, control; GABA, 5 mM GABA; Cd, 50 µM $CdCl_2$; GABA + Cd, 5 mM GABA and 50 µM $CdCl_2$ co-treatment. FW: fresh weight. Error bars show standard deviations (n = 3). Different lowercase letters indicate significant differences at a level of $P \leq$ 0.05.

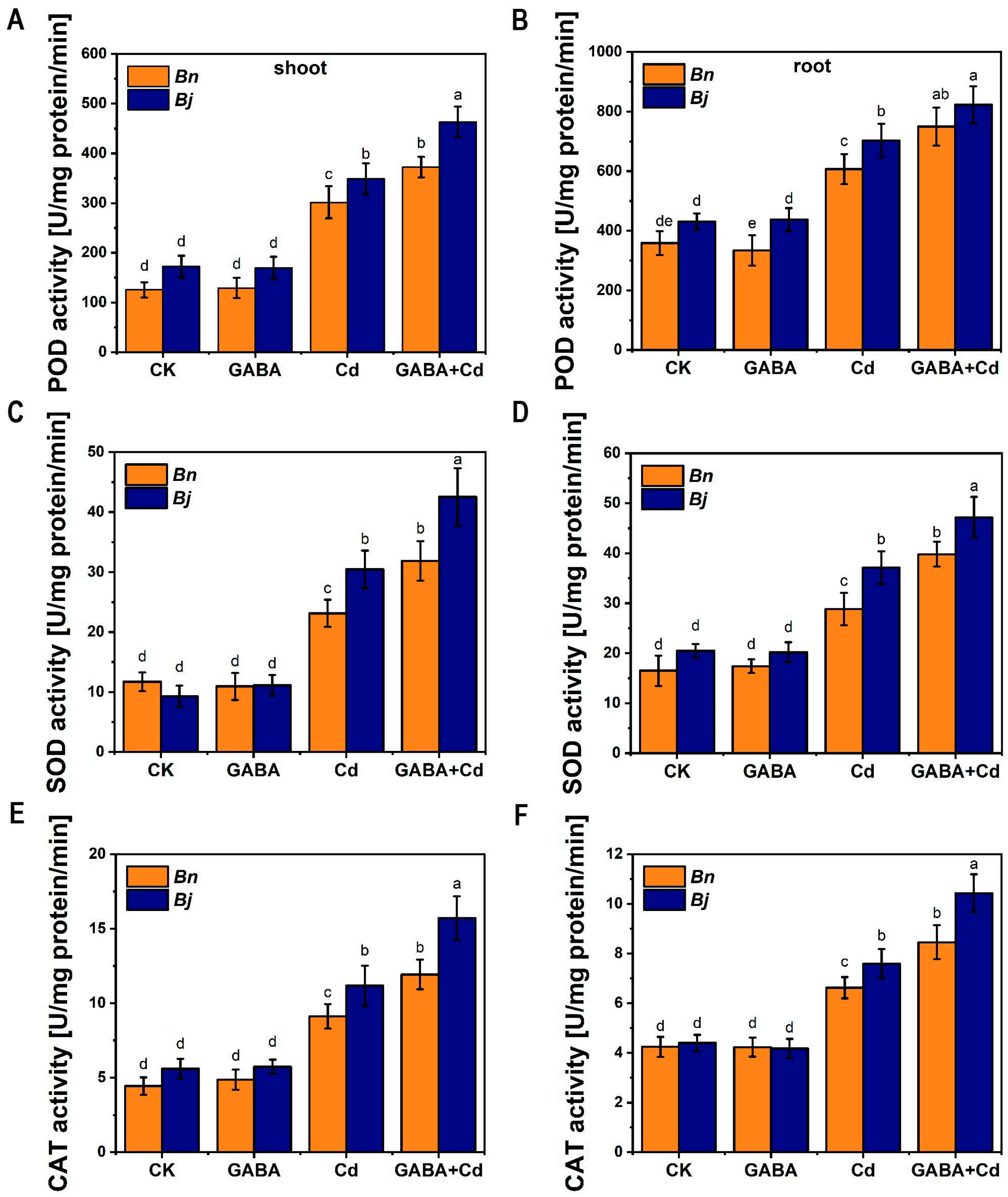

Supplementary Figure S3. Effects of GABA on antioxidant enzyme activity of *Brassica* speciesseedlings under Cd stress. POD activity in shoots (**A**) and roots (**B**). SOD activity in shoots (**C**) and roots (**D**). CAT activity in shoots (**E**) and roots (**F**). *Bn*, *Brassica napus*; *Bj*, *Brassica juncea*; CK, control; GABA, 5 mM GABA; Cd, 50 µM $CdCl_2$; GABA + Cd, 5 mM GABA and 50 µM $CdCl_2$ co-treatment. Error bars show standard deviations (n = 3). Different lowercase letters indicate significant differences at a level of $P \leq 0.05$.

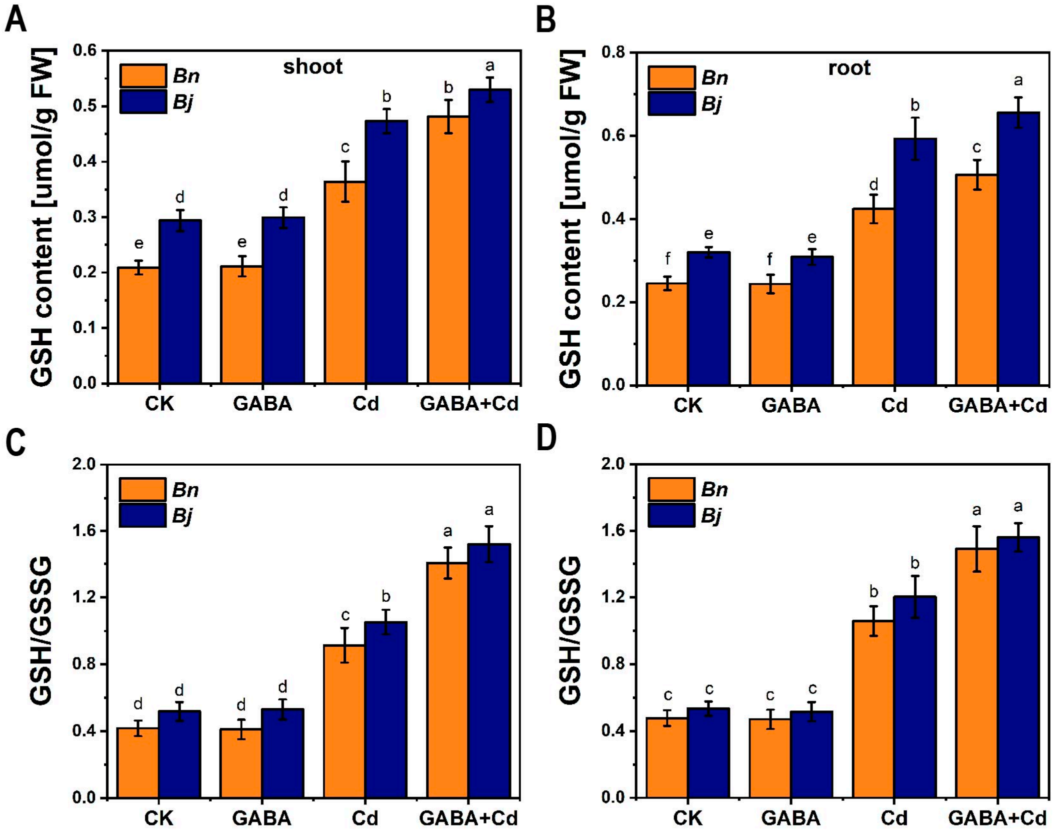

Supplementary Figure S4. Effects of GABA on GSH content and GSH / GSSG ratio in *Brassica* seedlings under Cd stress. GSH content in shoots (**A**) and roots (**B**). GSH / GSSG ratio in shoots (**C**) and roots (**D**). *Bn*, *Brassica napus*; *Bj*, *Brassica juncea*; CK, control; GABA, 5 mM GABA; Cd, 50 μM $CdCl_2$; GABA + Cd, 5 mM GABA and 50 μM $CdCl_2$ co-treatment. FW: fresh weight. Error bars show standard deviations (n = 3). Different lowercase letters indicate significant differences at a level of $P \leq 0.05$.

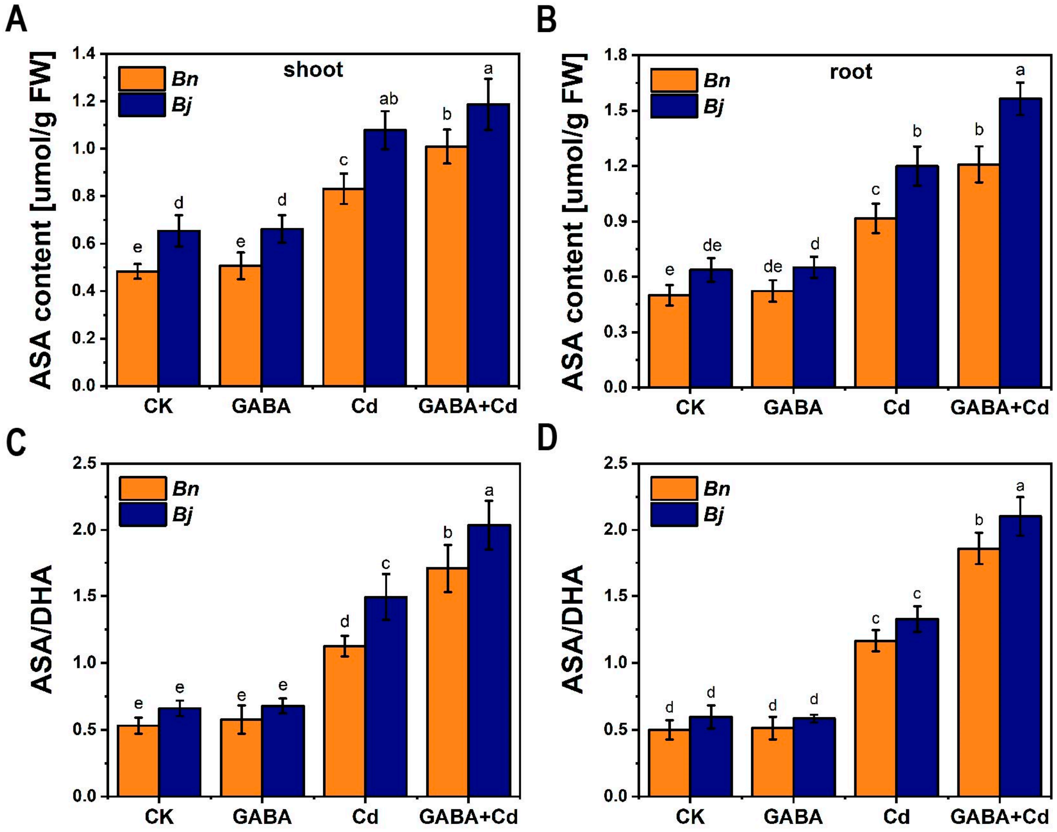

Supplementary Figure S5. Effects of GABA on ASA content and ASA / DHA ratio in *Brassica* seedlings under Cd stress. ASA content in shoots (**A**) and roots (**B**). ASA / DHA ratio in shoots (**C**) and roots (**D**). *Bn*, *Brassica napus*; *Bj*, *Brassica juncea*; CK, control; GABA, 5 mM GABA; Cd, 50 μM $CdCl_2$; GABA + Cd, 5 mM GABA and 50 μM $CdCl_2$ co-treatment. FW: fresh weight. Error bars show standard deviations (n = 3). Different lowercase letters indicate significant differences at a level of $P \leq 0.05$.
